# A Digital Software Support Platform for Hyperthyroidism Management in South Korea: Markov Simulation Model-Based Cost-Effectiveness Analysis

**DOI:** 10.2196/56738

**Published:** 2025-07-22

**Authors:** Jung Hyun Kim, Jaeyong Shin, Man S Kim, Jae Hoon Moon

**Affiliations:** 1 Division of Tourism and Wellness, Hankuk University of Foreign Studies, 81 Oedae-ro, Mohyeon-eup, Cheoin-gu, Yongin, 17035, Republic of Korea; 2 Institute for Innovation in Digital Healthcare, Yonsei University Health System, Seoul, Republic of Korea; 3 Department of Preventive Medicine, Yonsei University College of Medicine, Seoul, Republic of Korea; 4 Translational-Transdisciplinary Research Center, Clinical Research Institute, Kyung Hee University Hospital at Gangdong, School of Medicine, Kyung Hee University, Seoul, Republic of Korea; 5 Center for Artificial Intelligence in Healthcare, Seoul National University Bundang Hospital, Seongnam, Republic of Korea; 6 THYROSCOPE INC, Ulsan, Republic of Korea; 7 Department of Internal Medicine, Seoul National University Bundang Hospital, Seoul National University College of Medicine, 82, Gumi-ro 173 Beon-gil, Bundang-guSeongnam-si, Gyeonggi-do, 13620, Republic of Korea, 82-31-787-7068

**Keywords:** hyperthyroidism, digital monitoring solution, wearable devices, mobile-based monitoring, cost-effectiveness analysis, QALYs, hyperthyroidism management, apps, applications, digital health, digital technology, digital interventions, SDG3:good health and well-being, quality-adjusted life years

## Abstract

**Background:**

The integration of wearable technology for heart rate monitoring offers potential advancements in managing hyperthyroidism by providing a feasible way to track thyroid function. Although digital health solutions are gaining traction in various chronic conditions, their cost-effectiveness in hyperthyroidism management requires deeper investigation.

**Objective:**

This study aimed to evaluate the cost-effectiveness of a wearable or mobile-based thyroid function digital monitoring solution for hyperthyroidism management and to make a comparison with the existing standard approach within the South Korean health care context.

**Methods:**

We developed a decision-analytic Markov microsimulation model to simulate the cost and effectiveness of digital monitoring in a cohort of 10,000 hypothetical hyperthyroidism patients aged 40 years. The analysis was conducted from the perspective of the health care system, with a 4.5% annual discount rate applied to costs and effectiveness and an inflation adjustment to 2022 values. Model inputs were sourced from clinical studies, publicly available datasets, and expert input, with outcomes measured in quality-adjusted life years (QALYs). Cost-effectiveness was evaluated through incremental cost-effectiveness ratios (ICERs) and net monetary benefits (NMB), with additional deterministic and probabilistic sensitivity analyses performed to address input uncertainties.

**Results:**

Integrating digital monitoring yielded an additional 0.32 QALYs per patient at an incremental cost of US $3143, resulting in an ICER of US $9804.30 per QALY, significantly below the South Korean willingness-to-pay threshold of US $32,255/QALY. The digitally supported group exhibited improved rates of long-term remission (22.68%, 2268/10,000) and reduced postremission relapse (17.87%, 1787/10,000) compared to standard care (17.48%, 1748/10,000 and 26.37%, 2637/10,000, respectively). Probabilistic sensitivity analysis showed that digital intervention was the preferred cost-effective strategy in 64.4% (6440/10,000) of iterations. Subscription costs of the digital platform and the utility weight for thyroid-associated orbitopathy emerged as key factors affecting the ICER in sensitivity analyses.

**Conclusions:**

The findings suggest that digital monitoring provides a cost-effective strategy for enhancing hyperthyroidism management, supporting sustained remission, and reducing relapse rates. As such, digital solutions could serve as a valuable adjunct to traditional care, with the cost-effectiveness analysis providing an economic basis for determining pricing and value-based reimbursement in health care systems. The study underscores the importance of integrating digital solutions in chronic disease management and suggests that further research should include societal costs, such as productivity, to capture economic benefits fully.

## Introduction

An overactive thyroid, medically termed hyperthyroidism or thyrotoxicosis, occurs when the thyroid gland generates an excessive amount of thyroid hormones. Graves disease (GD) is the most common etiology for hyperthyroidism, with an estimated annual incidence ranging from 20 to 50 cases per 100,000 persons [1]. In 2018, the prevalence of hyperthyroidism was 0.25%, and the age-standardized incidence in South Korea was 40.3 and 105.5 per 100,000 men and women, respectively [2]. Hyperthyroidism leads to a range of symptoms and signs due to thyroid hormones’ impact on multiple organs. These include fatigue, anxiety, palpitations, excessive sweating, intolerance to heat, disrupted sleep, tremor, diarrhea, and weight loss [3]. Hyperthyroidism can be diagnosed through a thyroid function test (TFT) that measures serum thyroid hormone concentrations, including free thyroxine, thyrotropin, and other clinical factors. However, due to the nonspecific symptoms of hyperthyroidism, the timing of TFTs is often delayed, resulting in the diagnosis of hyperthyroidism occurring after it has significantly progressed. Therefore, having a straightforward and easily measurable parameter to predict thyroid function status would prove beneficial for the management of hyperthyroidism.

Photoplethysmography uses infrared light to monitor changes in blood circulation volume. This measurement offers valuable insights into the functioning of the cardiovascular system [4]. In recent times, the use of photoplethysmography technology for heart rate monitoring has gained popularity, primarily because it is easy to use and comfortable for wearers [5]. Various studies assessing the precision of wrist-worn heart rate monitors have been conducted, demonstrating relatively accurate heart rate measurements, especially for resting heart rate [6,7]. Collecting detailed longitudinal heart rate data and physical activity information using these wearable devices is straightforward, and such data can offer more insights than sporadic heart rate measurements. This capability enables the collection and analysis of more comprehensive and precise heart rate data, a crucial parameter that is affected by thyroid function status [8,9]. A medical software platform is under development that uses wearable technology to predict thyroid function state and assist in the management of thyroid dysfunction. It includes a mobile app and web solution for this purpose and is currently pending regulatory approval.

**Figure 1.**
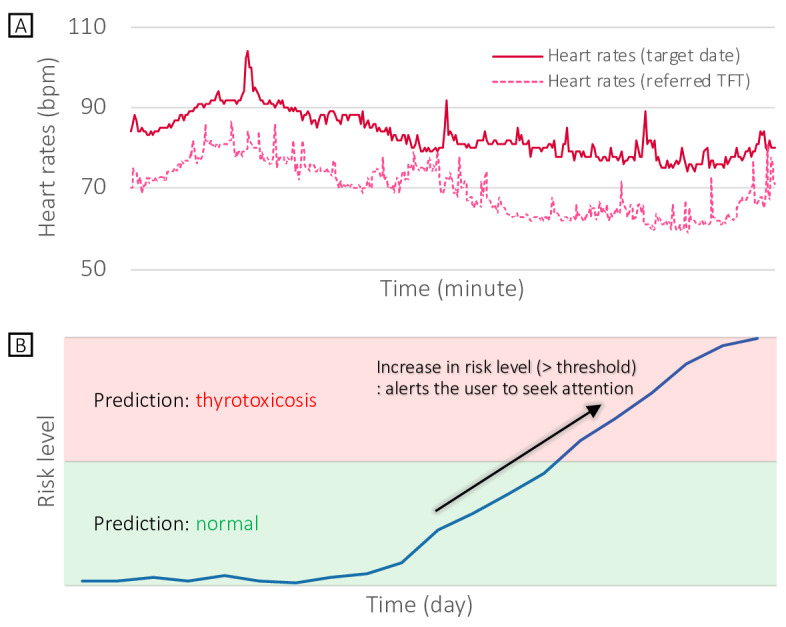
Description of a wearable or mobile-based thyroid function digital monitoring solution designed for the effective management of hyperthyroidism. (A) Heart rates collected from a wearable device on each date (the date of referred thyroid function test and target date), and (B) the risk level of hyperthyroidism calculated for the target date is determined by comparing the heart rate distribution over N days, including the target date, with the heart rate distribution over N days, including the day of the referred thyroid function test. In cases where the risk level surpasses the designated threshold, a notification is sent to the user, prompting them to seek medical attention. TFT: thyroid function test.

Despite the promising potential of wearable technology for managing thyroid dysfunction, there is limited research published on its actual impact concerning health care outcomes and overall costs. Therefore, we aimed to assess the cost-effectiveness of introducing a wearable or mobile-based thyroid function digital monitoring solution for hyperthyroidism management (Figure 1) driven by heart rate monitors commercialized as digital therapeutics and to compare it with the current standard approach.

## Methods

### Model Overview

**Figure 2.**
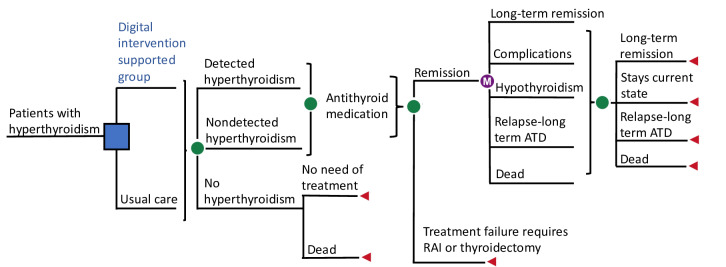
A basic description of the structure in the decision analytic Markov model. ATD: antithyroid drug; RAl: radioactive iodine.

We constructed a decision-analytic Markov microsimulation model to simulate 10,000 iterations of the clinical course of patients with hyperthyroidism (Figure 2). While a strict definition distinguishes between hyperthyroidism and GD, this paper refers to hyperthyroidism and conducts data analysis under this terminology. However, in South Korea, the incidence of toxic nodules as the underlying cause of hyperthyroidism is exceedingly low, accounting for less than 0.5% of cases [10]. As a result, patient management in this study followed a protocol for GD. This model consists of several health states in which the simulated individual can be in and move between. The statistical definition of the model is a discrete-time stochastic process with the Markov property [11]. The baseline cohort was comprised of 40-year-old individual patients with hyperthyroidism in South Korea. To advance time in our model, we used an annual cycle length, and the simulation continued until death or 100 years of age. The simulation of the natural disease progression and the effect of screening in men and women requires data including prevalence, incidence, risk of events, morbidity, and mortality. The model captures the variability in individual patient responses to treatment and disease progression over time. Each iteration represents a single hypothetical patient moving through various health states based on probabilistic transitions, including remission, long-term remission, complications, hypothyroidism, relapse, long-term antithyroid drug (ATD) use, and death. Some proportion of patients remain in their current state (hypothyroid state), while others may transition to different health states, such as long-term remission or relapse requiring long-term ATD therapy to capture more realistic transitions between health states and better reflect the long-term impacts of treatments. Eventually, patients in any state could transition to an absorbing state, such as death. A lifetime Markov model was simulated over a total of 70 cycles with a one-year cycle length. Quality-adjusted life years (QALYs), which include both quantity (life-years gained) and quality (health-related quality of life incremental cost-effectiveness in utility value), were used as effectiveness variables. Incremental cost-effectiveness ratio (ICER) and incremental net monetary benefit were calculated to evaluate cost-effectiveness. By aggregating the outcomes from all 10,000 iterations, we estimate the overall clinical and economic impact of different strategies for hyperthyroidism. Figure 2 depicts the core model, and the concept of combining time variables, treatment eligibility, and clinical prognosis was adapted from the previous studies.

### National Health Insurance Service Claims Data

A retrospective, population-based cohort study was conducted to investigate complications associated with hyperthyroidism and the related costs in Korea. The study examined enrollment and claims data from individuals covered by the National Health Insurance Service (NHIS) between 2003 and 2015. Patients with hyperthyroidism were defined as those who had 2 or more diagnostic codes of E05 or E06 and had been prescribed ATDs for at least 6 months. These drugs included MMI (191801ATB and 191802ATB), carbimazole (471501ATB, 471502ATB, and 471503ATB), and PTU (220101ATB and 220102ATB). The final cohort consisted of 1171 patients, excluding those who had taken medications for less than 6 months, were younger than 20 years, or had missing information (Multimedia Appendix 1). The major complications considered were thyroid-associated orbitopathy (TAO), heart failure, atrial fibrillation, osteoporosis, fractures, and thyroid cancer. Direct medical costs, including physician and hospital reimbursements for treatment procedures, as well as the annual mean cost for each complication, were obtained from NHIS data (Table 1).

**Table 1.** Base case values and references of model input parameters.

Parameter	Estimation	Distribution	Source
Prevalence of hyperthyroidism	7.20% (range 5.76%-8.64%)	Beta	[12]
Failure of ATD^a^	5% (range 4%-6%) over 1.5 years	Beta	[13]
Hypothyroidism	2.9% (range 2.32%-3.48%) over 10.2 years	Beta	[14]
Relapse postremission with ATD	52.8% (range 42.24%-63.36%) over 3.73 years (reverts to 0 after 5 years)	Beta	[15]
Thyroid-associated orbitopathy	25% (range 20%-30%)	Beta	[16]
Atrial fibrillation	4.3% (range 3%-5%)	Beta	NHIS^b^ [17],
Heart failure	1.6% (range 1.1%‐2.1%)	Beta	NHIS
Osteoporosis	1.59% (range 1.09%‐2.09%)	Beta	NHIS
Fracture	3.3% (range 2.8%‐3.8%)	Beta	NHIS
Thyroid cancer	1.23% (range 0.9%‐2%)	Beta	[18]
Hyperthyroidism sensitivity	87.13 (range 78.4‐95.8)	Beta	GlandyTM, THYROSCOPE INC
Hyperthyroidism specificity	83.78 (range 75.4‐92.15)	Beta	GlandyTM
T4^c^ + TSH^d^ sensitivity	100	Uniform	[19], Assumption
T4 + TSH specificity	100	Uniform	[19], Assumption
QALYs^e^, estimate (range)			
Thyroid cancer	0.897 (0.852‐0.941)	Beta	[20]
Hypothyroid	0.9479 (0.902‐0.997)	Beta	[21]
Thyroid-associated orbitopathy	0.84 (0.798‐0.882)	Beta	[22]
Hyperthyroidism with ATD	0.86 (t0.817‐0.903)	Beta	[23]
Osteoporotic fracture	0.91 (0.8645‐0.9555)	Beta	[24]
Fracture	0.83 (0.7885‐0.8715)	Beta	[24]
Atrial fibrillation	0.81 (0.729‐0.891)	Beta	[25]
Heart failure	0.78 (0.702‐0.858)	Beta	[25]
Cost (US $), estimate (range)			
Acute care costs (initial year)	1125.15 (1012.64‐1237.67)	Gamma	NHIS
Thyroid-associated orbitopathy (annual)	386.77 (367.44‐406.11)	Gamma	NHIS
Atrial fibrillation (annual)	150.54 (143.02‐158.07)	Gamma	NHIS
Heart failure (annual)	439.63 (417.65‐461.61)	Gamma	NHIS
Osteoporosis (annual)	208.49 (198.07‐218.91)	Gamma	NHIS
Fracture (annual)	347.41 (330.04‐364.78)	Gamma	NHIS
Thyroid cancer (annual)	149.23 (141.77‐156.69)	Gamma	NHIS
Hypothyroidism (annual)	250.37 (225.34‐275.41)	Gamma	NHIS
Long-term ADT^f^ (annual)	48.87 (43.98‐53.76)	Gamma	NHIS
Additional cost		
Digital software aid use (subscription fee per month)	US $30 (40,000 KRW; range US $10-$50)^g^	Gamma	Assumption
TSH, free T4 test fee	US $92.44 (US $64.71‐$120.17)	Gamma	NHIS
Discount rates	4.5% (3%-5%)	—^h^	[26]

a ATD: antithyroid drug.

b NHIS: National Health Insurance Service.

c T4: thyroxine.

d TSH: thyroid-stimulating hormone.

e QALY: quality-adjusted life year.

f ADT: antithyroid drug therapy.

g KRW: Korean won.

h Not applicable.

### Intervention and Comparators

To identify the potential effects of the digital monitoring solution used for hyperthyroidism management, we compared 2 strategies in this study. In the control group, suspected patients with hyperthyroidism were diagnosed and treated with their usual care without using the digital solution, while the digitally supported group was assisted by the digital monitoring solution in the management and treatment of hyperthyroidism. We hypothesize that having a daily check of hormone status using this digital solution may lead to reduced hospital visits and an increase in the probability of long-term remission.

### Hyperthyroidism and Complications

In this analysis, we used the data from randomized controlled trial studies, the NHIS claims data [27], and the published literature for determining the probabilities of individuals with hyperthyroidism and its complications from the screening procedure. The primary objective of hyperthyroidism management was to limit the number of major complications, including TAO, heart failure, atrial fibrillation, osteoporosis, fractures, and thyroid cancer. Rates and probabilities of major complications were from a Korean setting were obtained from the NHIS and supplemented with data retrieved from the published literature based on the systematic review. Standard all-cause and disease-specific mortality based on age and risk for South Korea in 2021 [28] was used in the simulation model.

### Quality-of-Life Adjustments and Costs

Utilities were estimated for all major events, including hyperthyroidism and related complications, which were obtained from published literature based on a systematic review and meta-analysis. Utilities were derived from a patient perspective to provide a more accurate representation of quality-of-life impacts by reflecting patients’ lived experiences and to account for adaptation to chronic conditions over time. This context-specific approach yields more relevant data for specific treatments, especially for conditions with long-term quality-of-life implications, such as hyperthyroidism or hypothyroidism [29-32]. We adopted a health care system perspective according to the economic evaluation guidelines in Korea [26]. Direct medical care costs, including physician and hospital reimbursement for treatment procedures, were obtained from the NHIS. Total mean medical care costs for hyperthyroidism, TAO, heart failure, atrial fibrillation, osteoporosis, fractures, and thyroid cancer were obtained and confirmed from the published literature. Monthly subscription fees for the group with digital monitoring solution use were recurring annually and applied for a lifetime. Sensitivity analyses were performed to assess the association of cost reduction due to unnecessary hospital visits using the thyroid function digital monitoring solution (70%‐100%). A 4.5% discount rate was used for both costs and effects in the base-case scenario. Costs were adjusted to the year 2022 using consumer price indices for medical care [33] and converted to 2022 US dollars (US $1=1293.68 KRW) [34]. Table 1 presents the quality of life and utility decrements used in the model together with the other parameters.

### Statistical Analysis

Our primary endpoints were QALYs, total costs (2022 USD), and ICERs. Future costs and QALYs were discounted at a rate of 4.5%. We used a willingness-to-pay (WTP) threshold of US $32,255 per QALY gained, reflecting 1-time the gross domestic product per capita in 2022 South Korea [26,35-38] to determine cost-effectiveness. A strategy was considered preferred if it resulted in the greatest increase in QALYs while being cost-effective. Overall, this study followed the Consolidated Health Economic Evaluation Reporting Standards (CHEERS) guideline (Checklist 1) [39].

To assess how the uncertainty in model inputs influences the outcomes, we conducted 1-way and probabilistic sensitivity analyses. In 1-way sensitivity analyses, we systematically changed 1 parameter at a time within a specified range of values while keeping all other input variables constant at their base-case values. The upper and lower bounds for these parameter ranges were determined based on either the minimum and maximum values available in relevant databases or the range of 90%-110% for each value.

Probabilistic sensitivity analyses were performed by repeatedly sampling model inputs simultaneously from defined probabilistic distributions. We used gamma distributions for costs and beta distributions for all other parameters. This process involved running the model 10,000 times, considering a cohort of 10,000 patients. We then calculated the percentage of instances in which each strategy was favored under the WTP thresholds.

### Ethical Considerations

The procedures of this study underwent expedited review and received approval from the institutional review board of Severance Hospital, Yonsei University (IRB number: 4-2022-1525). In accordance with local legislation and institutional requirements, ethical approval was not mandatory for this study. Likewise, written informed consent for participation was not required, in accordance with national legislation and institutional requirements. All data have been deidentified.

## Results

### Base-Case Analysis

The results of base-case cost-effectiveness analysis are presented in Table 2. Patients with the implementation of the digital monitoring solution in managing hyperthyroidism were associated with increased costs and increased QALYs. The model predicted that the implementation of digital software in managing hyperthyroidism produced 11.48 QALYs and US $9593 expected costs compared to 11.16 QALYs and US $6445 generated by treatment as usual without digital software aid over a lifetime horizon. The introduction of the thyroid function digital monitoring solution would increase costs by US $3143 and prolong QALYs by 0.32. The ICER associated with hyperthyroidism with digital intervention management tool was US $9804.30 per QALY gained (Table 2), which was well below the hypothetical threshold value in Korea of US $32,255 per QALY gained.

**Figure 3.**
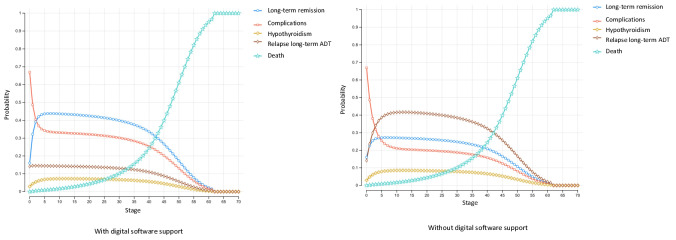
Markov probability analysis of each state with and without digital software support. ADT: antithyroid drug therapy.

Notably, the Markov probability analysis revealed that hyperthyroidism management with digital intervention increased the proportion of long-term remission and reduced the likelihood of postremission relapse of hyperthyroidism (Table 2 and Figure 3).

**Table 2.** Effectiveness and cost estimates for two strategies on a simulated cohort of patients with hyperthyroidism.

	Hyperthyroidism managed without digital software support	Hyperthyroidism managed with digital software support
Long-term remission, % (n/N)	17.48 (748/10,000)	22.68 (2268/10,000)
Complications, % (n/N)	15.17 (1517/10,000)	18.84 (1884/10,000)
Hypothyroidism, % (n/N)	5.40 (540/10,000)	5.02 (502/10,000)
Relapse postremission with ATD^a^, % (n/N)	26.37 (2637/10,000)	17.87 (1787/10,000)
Discounted lifetime cost per person, US $	6449.77	9592.55
Discounted incremental cost per person, US $	—	3142.78
Discounted lifetime effectiveness per person, QALY^b^	11.16	11.48
Discounted incremental effectiveness per person, QALY	—	0.32
Discounted ICER^c^, incremental costs per QALY gained	—	9804.30
Discounted NMB^d^ in WTP^e^ of US $32,255, US $	436,952,569.9	449,505,659.6
Discounted INMB^f^ in WTP of US $32,255, US $	—	12,553,089.7

a ATD: antithyroid drug.

b QALY: quality-adjusted life year.

c ICER: incremental cost-effectiveness ratio.

d NMB: net monetary benefit.

e WTP: willingness to pay.

f INMB: incremental net monetary benefit.

### Sensitivity Analysis

**Figure 4.**
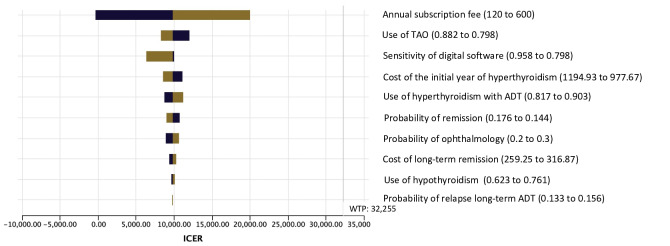
Tornado diagram for incremental cost-effectiveness ratio. ADT: antithyroid drug therapy; ICER: incremental cost-effectiveness ratio; TAO: thyroid-associated orbitopathy; WTP: willingness to pay.

We conducted a 1-way deterministic sensitivity analysis. The results of the 1-way deterministic sensitivity analyses with the tornado diagram are shown in Figure 4. The annual cost of the digital monitoring solution use, which ranged from US $120 to US $600, had the largest impact on the ICER value, followed by use of TAO and the sensitivity of the digital software. According to the one-way threshold analysis for the monthly digital support cost, the digital software aid arm became the dominant strategy when the cost was below US $10 per month (Multimedia Appendix 1 and Figure 2). Even the highest ICER value observed in this diagram was still below the threshold value of US $32,255 per QALY gained.

In Table 3, the ICERs were altered based on the assumption that hospital visits for TFT would decrease as hormone status is monitored and managed. This reduction would lead to lower costs associated with unnecessary hospital visits and tests.

**Figure 5.**
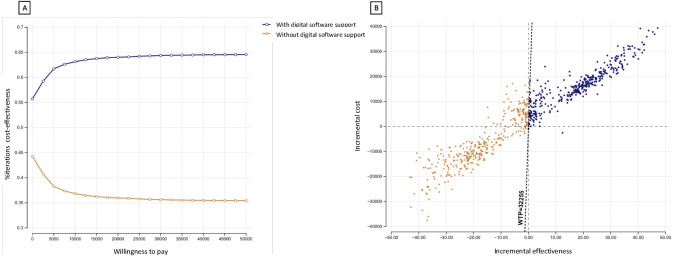
Results of probabilistic sensitivity analysis: (**A**) cost-effectiveness acceptability curve and (**B**) incremental cost-effectiveness scatterplot. WTP: willingness to pay.

For the Monte Carlo simulations, we presented the results of probabilistic sensitivity analysis as a cost-effectiveness acceptability curve, cost-effectiveness scatter plot, and incremental cost-effectiveness scatterplots. To study the uncertainty in our results, the statistical uncertainty was tested probabilistically 10,000 times. The result of the probabilistic analysis presented as acceptability curves in Figure 5 described the probabilities of being cost-effective when WTP changes. From the results of the Monte Carlo simulation, the digital intervention was the optimal cost-effective strategy with a probability of 64.4% represented in the form of an ICE scatterplot in Figure 5.

**Table 3.** One-way sensitivity analysis by reduction of cost for hospital visits.

Strategy	Cost (US $)	QALY^a^ (years)	ICER^b^ ($ per QALY)
Base case			
Treatment as usual	6449.77	11.16	—
Management with digital aid	9592.56	11.48	9804.31
10% cost reduction from unnecessary hospital visits			
Treatment as usual	6449.77	11.16	—
Management with digital aid	9541.50	11.48	9654.04
20% cost reduction from unnecessary hospital visits			
Treatment as usual	6449.77	11.16	—
Management with digital aid	9490.45	11.48	9485.76
30% cost reduction from unnecessary hospital visits			
Treatment as usual	6449.77	11.16	—
Management with digital aid	9439.39	11.48	9326.48

a QALY: quality-adjusted life year.

b ICER: incremental cost-effectiveness ratio.

## Discussion

### Principal Findings

The application of artificial intelligence–driven wearable devices in cardiac disease management is operative; however, there is little evidence of their applications in thyroid disease and its cost-effectiveness. To the best of our knowledge, this is the first economic evaluation that considers the cost-effectiveness of a wearable or mobile-based thyroid function digital monitoring solution compared with standard care from a health care system perspective. The results demonstrated that this digital monitoring support would provide an incremental health gain per patient of 0.32 QALY with an ICER of US $9804.30 per QALY gained. A Markov microsimulation model was developed to optimize the use of data from the published studies based on a systematic review while accounting for the diversity and variability among patients with hyperthyroidism. The findings indicated that aiding patients with the digital monitoring solution resulted in slightly increased health care costs and improved health benefits compared to standard care. Therefore, the digital solution was considered highly cost-effective for functional outcomes in hyperthyroid management using a WTP threshold of US $32,255 per QALY gained.

Both deterministic and probabilistic sensitivity analyses demonstrated the robustness of our results. The cost-effectiveness was sensitive to the annual subscription fee for the hyperthyroidism management platform use, use of TAO, sensitivity of the digital device, initial treatment cost of hyperthyroidism, and use of long-term ATD. Despite these sensitivities, the digital intervention strategy maintained its advantageous position by including most of the parameters, with an ICER remaining below the threshold of US $32,255.

Our analysis revealed that the monthly cost of a digital monitoring solution for hyperthyroidism had the greatest impact on ICERs in the 1-way deterministic sensitivity analysis. Although the model inputs and target disease were different, other studies pointed out a similar conclusion by evaluating the cost-effectiveness of digital therapeutics for low back pain [40] and hypertension [41]. The result from our model indicates that even minor adjustments in pricing can shift the balance between different strategies in this particular scenario, highlighting the sensitivity of the economic implications of these digital tools to various factors such as implementation details, settings, payer viewpoints, and assumed unnecessary hospital visit reduction. Further research exploring diverse payment approaches for digital intervention will be essential for conducting robust comparisons and reaching definitive conclusions regarding the health economic outcomes linked to digital technology. In Korea’s single-payer system, the adoption of artificial intelligence–based systems is significantly influenced by the rate at which reimbursements are provided.

The major drawback of ATD therapy is the high recurrence rate of GD, the most common cause of hyperthyroidism, ranging from 20% to 70%, once the conventional 12‐18 months of treatment is discontinued [42]. The serum concentration of thyrotropin receptor antibodies, a significant predictor of relapse in GD, can fluctuate or remain elevated in patients with GD, regardless of the continuous use of ATD. Therefore, there is a higher incidence of GD recurrence in clinical remission states following ATD treatment. The median duration of remission after ATD treatment is reported to be approximately 6.8 years [43], leading to suggestions of continuous, long-term ATD therapy [44-47]. A meta-analysis has shown that for each additional year of ATD treatment beyond 24 months, the remission rate increases by 16% [48]. These findings suggest that in cases where thyrotropin receptor antibody levels fail to return to normal after the conventional 12‐18 months of ATD treatment, the possibility of considering long-term therapy should be explored [49]. Therefore, international medical societies are now considering long-term treatment as an option for managing Graves hyperthyroidism [42,50].

Significant adherence to pharmacological treatments is a multifaceted behavior influenced by various factors at different stages of a person’s medication journey. Adherence to long-term therapy for chronic illnesses is estimated to reach only 50% on average [51]. At present, no single intervention has proven consistently effective in addressing long-term nonadherence. Digital interventions for medication adherence, however, offer a potential solution to enhance adherence by providing multifaceted interventions tailored to individual needs. Digital technological interventions have displayed initial promise in boosting medication adherence rates and offering health care professionals the ability to monitor and assess adherence [52-55]. Effectively managing hyperthyroidism requires patients to integrate it into their daily lives. By integrating digital monitoring solutions into the management of thyroid dysfunction, patients can regularly check their thyroid function status as part of their daily life, ensuring adherence to their medication schedule and promoting long-term health outcomes improvement.

In this research, we demonstrated the implementation of a digital intervention aid specifically designed to aid in the management of patients with hyperthyroidism. This innovation holds the potential to enhance health care outcomes and deliver cost-effectiveness on a large scale. Our sensitivity analyses have highlighted the robustness of our findings, even when considering variations in model parameters and assumptions. It is important to note that our study primarily adopts the perspective of the Korean national health care system and does not encompass a broader view of societal costs. Given the fact that hyperthyroidism is prevalent among individuals younger than 65 years, it is conceivable that the cost-effectiveness associated with hyperthyroidism could be even more substantial if we were to take into account factors such as productivity losses and a wider range of societal costs [12].

### Limitations

This study comes with several limitations. We applied patient-derived uses in the study. Patients may underestimate the severity of certain health states due to adaptation or limited awareness of alternative health states, which can lead to higher-than-expected utility values. This effect is especially relevant in chronic conditions, where patients may grow accustomed to their symptoms over time; however, we adjusted these values by applying a decrement. While our analysis focused on the perspective of health care providers, it is essential to recognize that thyroid dysfunction has substantial health-related and economic implications not only for individual patients but also for society as a whole. The broader societal costs are expected to increase in the future, primarily due to the population with hyperthyroidism being an active working-age population in the society. In addition, because digital intervention–supported management has not been systematically integrated into health care practice in Korea and due to uncertainties surrounding the application of digital health frameworks, there remain ambiguities related to our estimates. Moreover, uncertainties are compounded by the possibility of negotiations influencing unit prices, which contribute to the overall expenses of the digital-based platform, especially if the intervention were to be introduced. We did not apply attrition rates in the model as we assumed that the patient with digital intervention would keep using the tool to manage hyperthyroidism, which could impact lifetime ICER. In addition, a limitation of this study is the potential discomfort associated with wearable or mobile-based monitoring solutions, particularly the burden of continuous use. This is especially relevant to older adult populations, who may face challenges with digital interventions, potentially adding a societal burden. Although capturing this discomfort in terms of costs or utility values is challenging, it may impact cost-effectiveness by affecting adherence and patient satisfaction. Future research should consider these factors, as they could influence the overall effectiveness and acceptability of digital health interventions.

### Conclusions

In conclusion, the integration of a wearable or mobile-based thyroid function digital monitoring solution demonstrated good cost-effectiveness in hyperthyroidism management compared to ATD treatment as usual. Digital monitoring solution cost and its sensitivity influence cost-effectiveness, and we need to explore the balance among the digital monitoring support application cost, accuracy of hormonal level change status, and effectiveness for target patients with hyperthyroidism in clinical practice. Digital therapeutics are currently in a pretest period to determine the extent of reimbursement they may receive from Korea’s national universal health insurance system. Therefore, evaluating the cost-effectiveness of digital interventions, as illustrated in this study, is gaining significance in establishing suitable pricing for value-based reimbursement.

## Supplementary material

10.2196/56738 Multimedia Appendix 1 Additional files.

10.2196/56738 Checklist 1 CHEERS (Consolidated Health Economic Evaluation Reporting Standards) 2022 checklist.

## Abbreviations

ATD: antithyroid drug
CHEERS: Consolidated Health Economic Evaluation Reporting Standards
GD: Graves disease
ICER: incremental cost-effectiveness ratio
NHIS: National Health Insurance Service
QALY: quality-adjusted life year
TAO: thyroid-associated orbitopathy
TFT: thyroid function test
WTP: willingness to pay
