# Supplementary material for: A Digital Software Support Platform for Hyperthyroidism Management in South Korea: Markov Simulation Model-Based Cost-Effectiveness Analysis
Source: JMIR Mhealth Uhealth. 2025 Jul 22;13:e56738. doi: 10.2196/56738 (PMC12325118; doi:10.2196/56738)
Supplement: Multimedia Appendix 1 [file mhealth-v13-e56738-s001.docx]

**Appendix document:**

**Figure S1.** Flowchart of patients with hyperthyroidism from the National Health Insurance Service (NHIS) claims data.


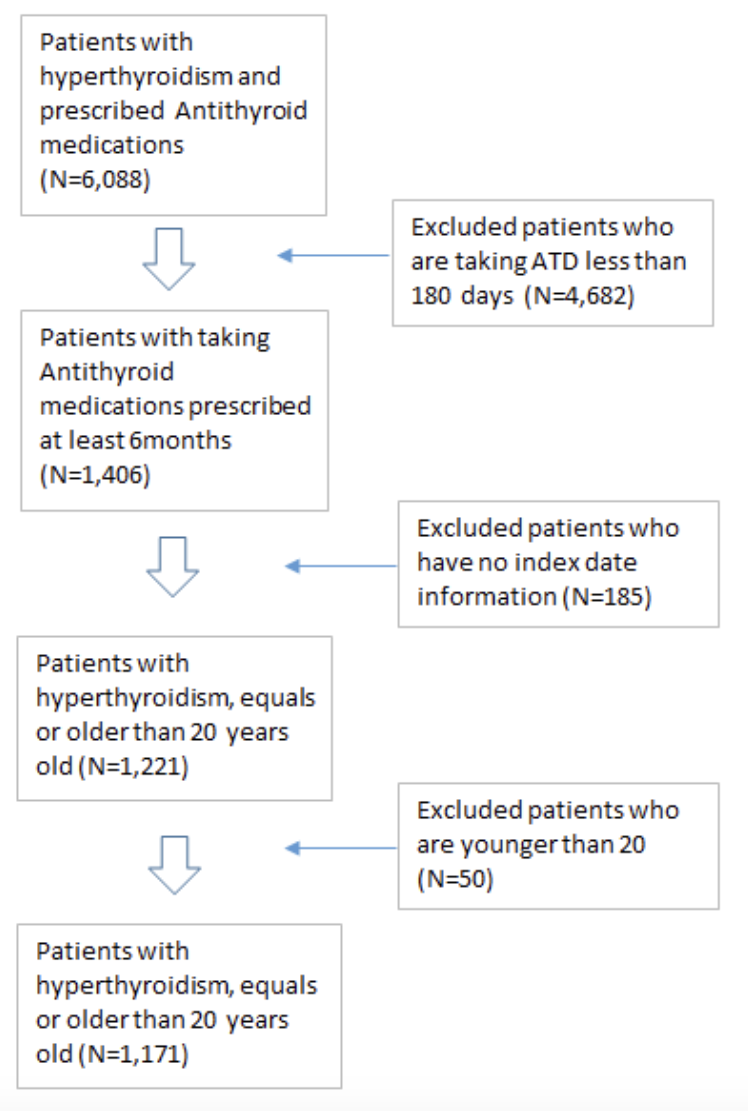


**Figure S2.** Results of deterministic one-way sensitivity analysis for changes in the annual subscription fee for hyperthyroidism management.
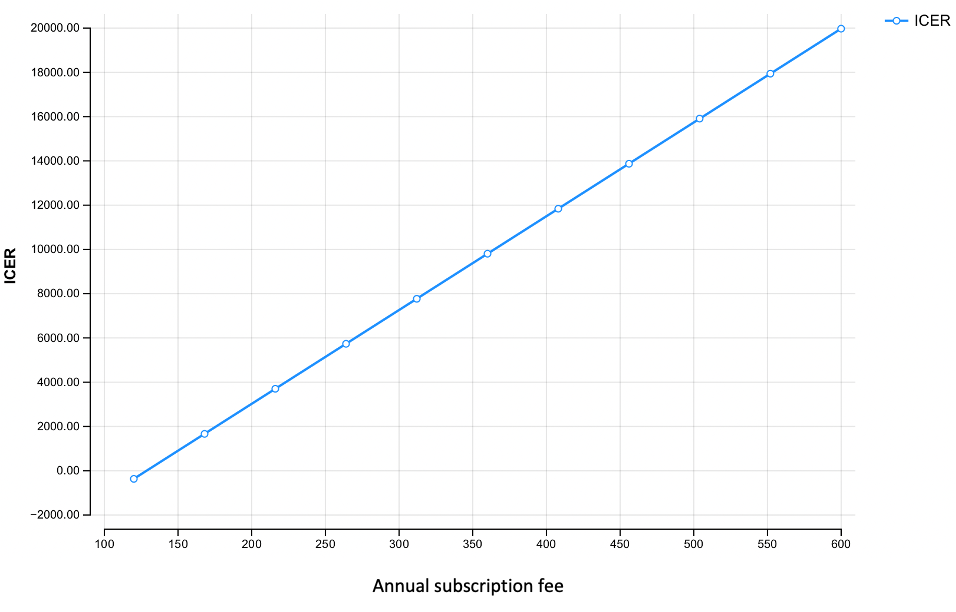


**Table S1.** Results of deterministic one-way sensitivity analysis by monthly (annual) subscription fee changes for hyperthyroidism management.

| Strategy | Monthly subscription fee (annual $) | Cost | Incr cost | Eff | Incr eff | ICER | |
| --- | --- | --- | --- | --- | --- | --- | --- |
| Treatment as usual |  | 6449.77 |  | 11.16 |  | |  |
| Management with digital aid | 10 (120) | 6332.49 | -117.28 | 11.48 | 0.32 | | -365.86 |
| Treatment as usual |  | 6449.77 |  | 11.16 |  | |  |
| Management with digital aid | 14 (168) | 6984.51 | 534.74 | 11.48 | 0.32 | | 1668.17 |
| Treatment as usual |  | 6449.77 |  | 11.16 |  | |  |
| Management with digital aid | 18 (216) | 7636.52 | 1186.75 | 11.48 | 0.32 | | 3702.20 |
| Treatment as usual |  | 6449.77 |  | 11.16 |  | |  |
| Management with digital aid | 22 (264) | 8288.53 | 1838.76 | 11.48 | 0.32 | | 5736.23 |
| Treatment as usual |  | 6449.77 |  | 11.16 |  | |  |
| Management with digital aid | 26 (312) | 8940.54 | 2490.77 | 11.48 | 0.32 | | 7770.27 |
| Treatment as usual |  | 6449.77 |  | 11.16 |  | |  |
| Management with digital aid | 30 (360) | 9592.55 | 3142.78 | 11.48 | 0.32 | | 9804.30 |
| Treatment as usual |  | 6449.77 |  | 11.16 |  | |  |
| Management with digital aid | 34 (408) | 10244.57 | 3794.80 | 11.48 | 0.32 | | 11838.33 |
| Treatment as usual |  | 6449.77 |  | 11.16 |  | |  |
| Management with digital aid | 38 (456) | 10896.58 | 4446.81 | 11.48 | 0.32 | | 13872.36 |
| Treatment as usual |  | 6449.77 |  | 11.16 |  | |  |
| Management with digital aid | 42 (504) | 11548.59 | 5098.82 | 11.48 | 0.32 | | 15906.39 |
| Treatment as usual |  | 6449.77 |  | 11.16 |  | |  |
| Management with digital aid | 46 (552) | 12200.60 | 5750.83 | 11.48 | 0.32 | | 17940.42 |
| Treatment as usual |  | 6449.77 |  | 11.16 |  | |  |
| Management with digital aid | 50 (600) | 12852.61 | 6402.84 | 11.48 | 0.32 | | 19974.45 |
